# Hereditary diffuse gastric cancer with carcinogenesis from ectopic gastric mucosa in the duodenum

**DOI:** 10.1007/s12328-025-02279-9

**Published:** 2026-01-27

**Authors:** Kazuki Horiuchi, Yusuke Ishibashi, Akira Tomioka, Kazuyuki Narimatsu, Hironori Tsujimoto, Ryota Hokari, Kei Kato

**Affiliations:** 1 Department of Internal Medicine, Japan Self-Defense Force Iruma Hospital, 2-1-4, Koyodai, Iruma, Saitama 358-0001 Japan; 2 Department of Surgery, Japan Self-Defense Force Iruma Hospital, 2-1-4, Koyodai, Iruma, Saitama 358-0001 Japan; 3 Department of Internal Medicine, National Defense Medical College, 3-2 Namiki, Tokorozawa, Saitama 359-8513 Japan; 4 Department of Surgery, National Defense Medical College, 3-2 Namiki, Tokorozawa, Saitama 359-8513 Japan; 5 Department of Pathology, Japan Self-Defense Force Iruma Hospital, 2-1-4, Koyodai, Iruma, Saitama 358-0001 Japan

**Keywords:** Hereditary diffuse gastric cancer, R732Q, Ectopic gastric mucosa

## Abstract

A 43-year-old man with a family history of gastric cancer had undergone endoscopic submucosal dissection (ESD) for a signet-ring cell carcinoma (SRCC) lesion. Five years later, a recurrent SRCC lesion was treated with a second ESD. Two months later, multiple recurrent SRCC lesions were again detected. Immunohistochemical staining revealed reduced E-cadherin expression. Genetic testing for *CDH1* identified an R732Q missense mutation. The patient subsequently underwent total gastrectomy, which identified 22 intramucosal SRCC lesions. Notably, the most distal carcinoma was located in the duodenal bulb and appeared to arise from mucosa containing gastric fundic glands, gastric foveolar epithelium, CD10-positive cells, MUC2-positive cells, and Brunner’s glands, suggesting origin from ectopic gastric mucosa in the duodenum. To our knowledge, this is the first reported case of SRCC arising from ectopic gastric mucosa in patients with hereditary diffuse gastric cancer. Screening for ectopic gastric mucosa may be warranted in patients with *CDH1* mutations.

## Introduction

Hereditary diffuse gastric cancer (HDGC) is an autosomal dominant inherited disease caused by *CDH1* mutations, leading to diffuse gastric cancer (DGC) due to abnormal expression of E-cadherin, a key cell adhesion protein [1]. According to the International Gastric Cancer Linkage Consortium (IGCLC) guidelines [2], total gastrectomy is the only curative treatment following a definitive diagnosis, as complete removal of the gastric mucosa is essential. Here, we report a case in which a patient underwent total gastrectomy after the identification of a *CDH1* mutation, with additional consideration of carcinogenesis arising from ectopic gastric mucosa in the duodenum.

## Case report

**Fig. 1:**
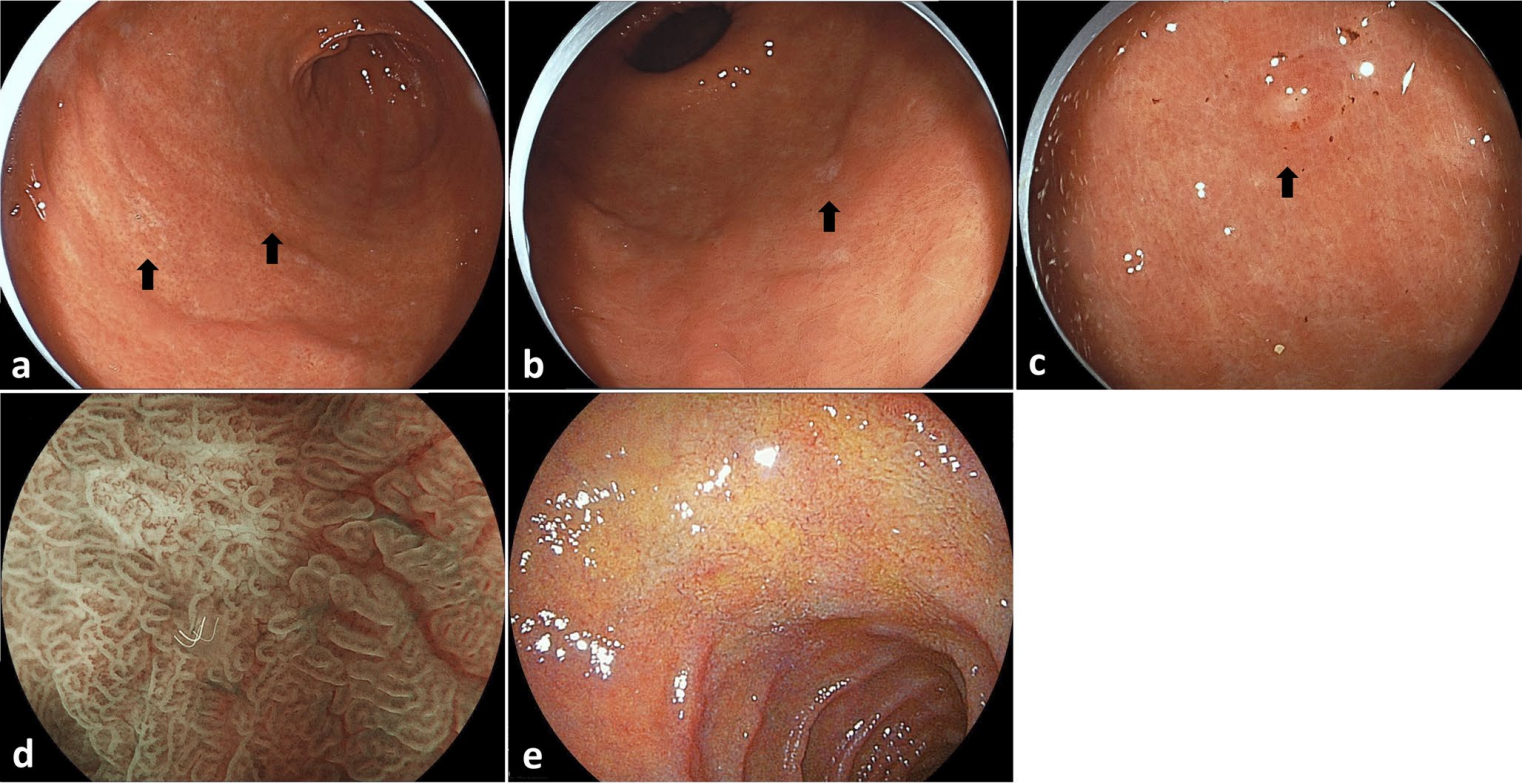
Representative images of esophagogastroduodenoscopy. Flat, whitish areas were observed on the anterior wall **a**, posterior wall **b**, and lesser curvature **c** of the antrum, as indicated by black arrows. All lesions were indicative of signet-ring cell carcinoma. Magnifying endoscopy with blue laser imaging **d** revealed irregular vascular patterns. **e** Linked color imaging of the duodenal bulb

**Fig. 2:**
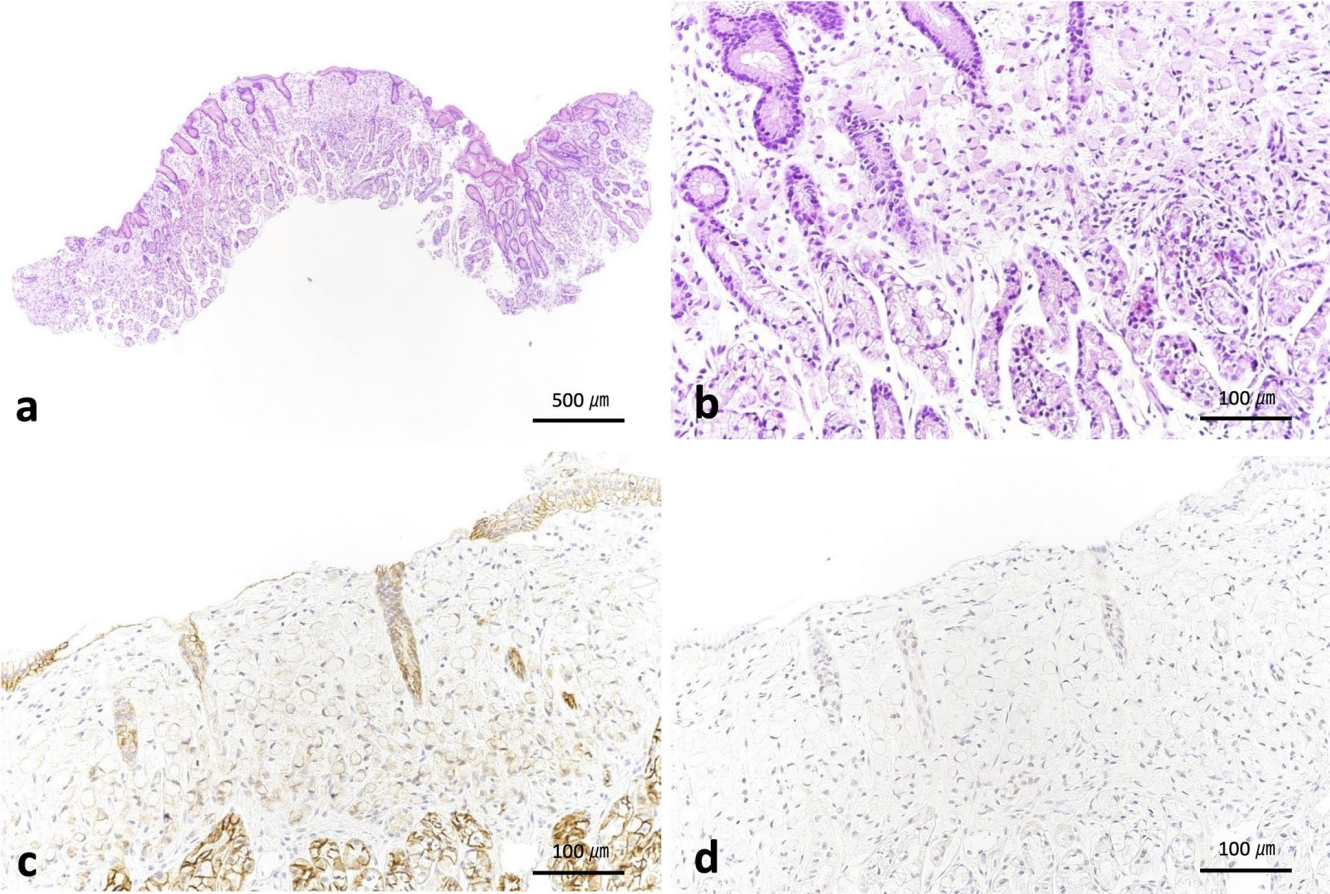
Pathological images of the biopsied specimen. **a** Hematoxylin and eosin–stained biopsy image showing signet-ring cell carcinoma (SRCC) in the squared area. **b** Enlarged view of SRCC. **c** Immunohistochemical staining for E-cadherin, showing reduced expression in SRCC. **d** Immunohistochemical staining for p53, showing no overexpression in SRCC

**Fig. 3:**
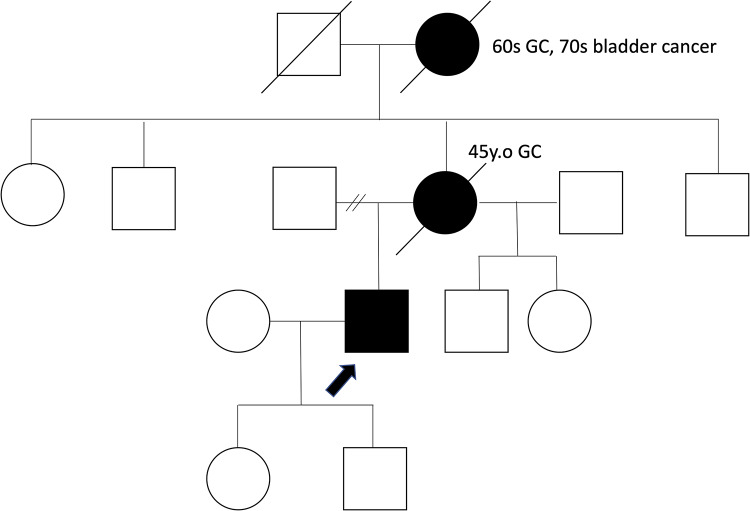
Family tree. His mother died of gastric cancer at age of 45, and his grandmother was diagnosed with gastric cancer in her 60s

A 43-year-old man underwent endoscopic submucosal dissection (ESD) after a 0–IIc lesion was incidentally identified on the anterior wall of the lower gastric body during esophagogastroduodenoscopy (EGD) at a routine medical checkup. Histological examination revealed signet-ring cell carcinoma (SRCC) confined to the mucosa. The patient underwent EGD follow-up every six months and showed no recurrence after ESD. At the age of 48, the patient began follow-up at our hospital. Initial EGD revealed a 0–IIc lesion in the fornix, which was biopsied and diagnosed as SRCC. The patient underwent ESD again, and pathological examination confirmed intramucosal carcinoma. Two months later, follow-up EGD revealed four whitish areas in the gastric antrum (Fig. 1), all of which were diagnosed as SRCC, and showing reduced E-cadherin expression and no p53 overexpression on biopsy (Fig. 2). We retrospectively reviewed the endoscopic images and did not identify any lesions in the antrum. Serum antibody testing and the urea breath test for *Helicobacter pylori* (*H.pylori*) were negative, and the biopsy specimen was also negative. There was no history of prior eradication therapy, with the patient being diagnosed as *H.pylori*-negative. Given the patient’s family history of gastric cancer (Fig. 3) and the recurrence of gastric cancer before the age of 50, we suspected hereditary diffuse gastric cancer (HDGC), although histological confirmation of gastric cancer in the patient’s relatives was not obtained.

**Fig. 4:**
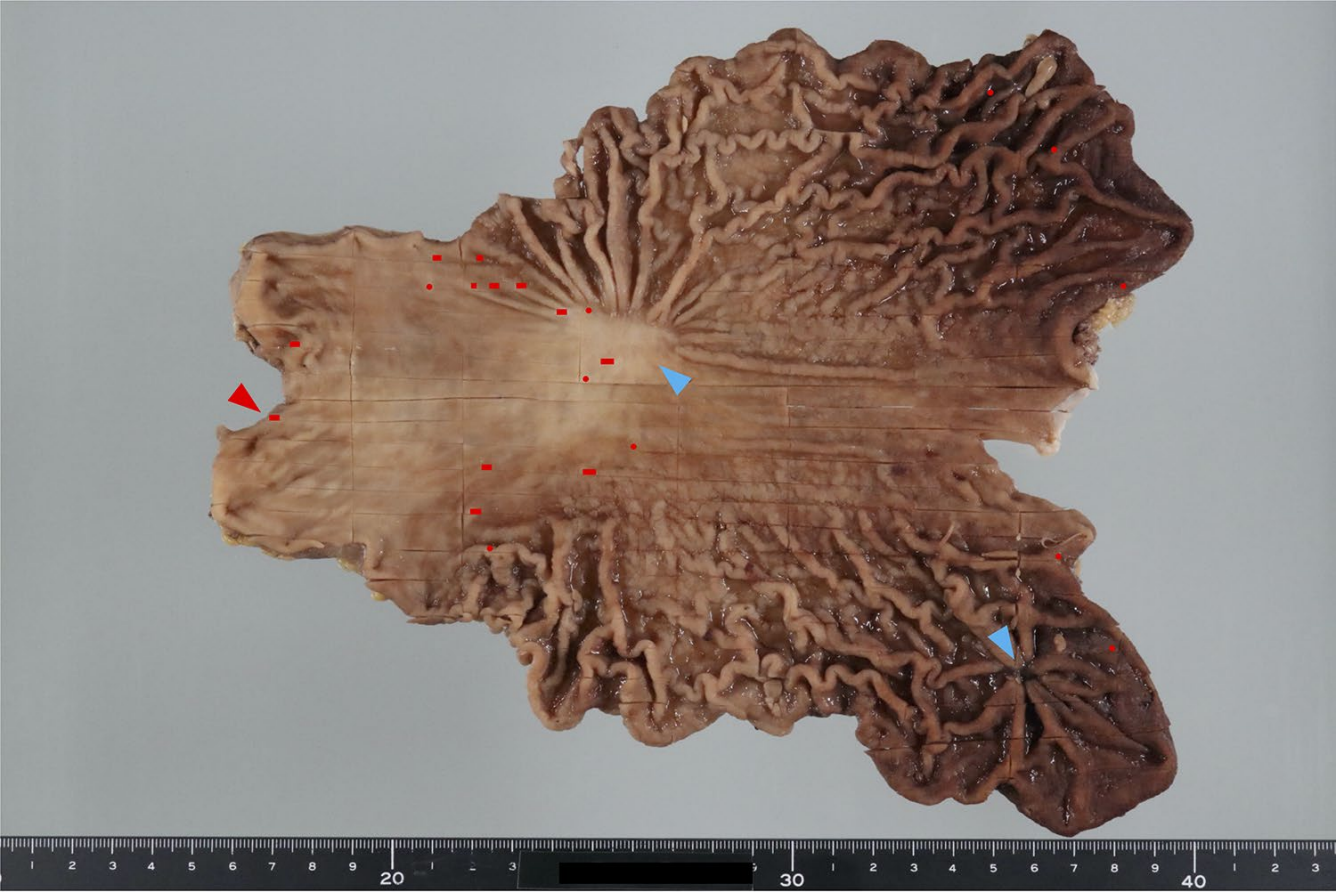
Gross appearance of the formalin-fixed stomach. The mapping indicates the location of 22 SRCC foci (red bars or red dots), one of which is the SRCC lesion arising from ectopic gastric mucosa in the duodenum (red arrowhead). Blue arrowheads indicate ESD scars

After obtaining consent, we conducted *CDH1* genetic testing, which identified the R732Q mutation. Given these findings, we recommended total gastrectomy, and upon receiving consent, we performed laparoscopic total gastrectomy with D1 lymph node dissection. Histopathological examination revealed 22 intramucosal SRCC lesions, measuring up to 2.5 mm in size, distributed throughout the stomach (Fig. 4). No vascular or lymphatic invasion was observed. No SRCC was identified at either the proximal or distal margin; however, the distance to the distal margin was 1 mm. No lymph node metastasis was observed.

**Fig. 5:**
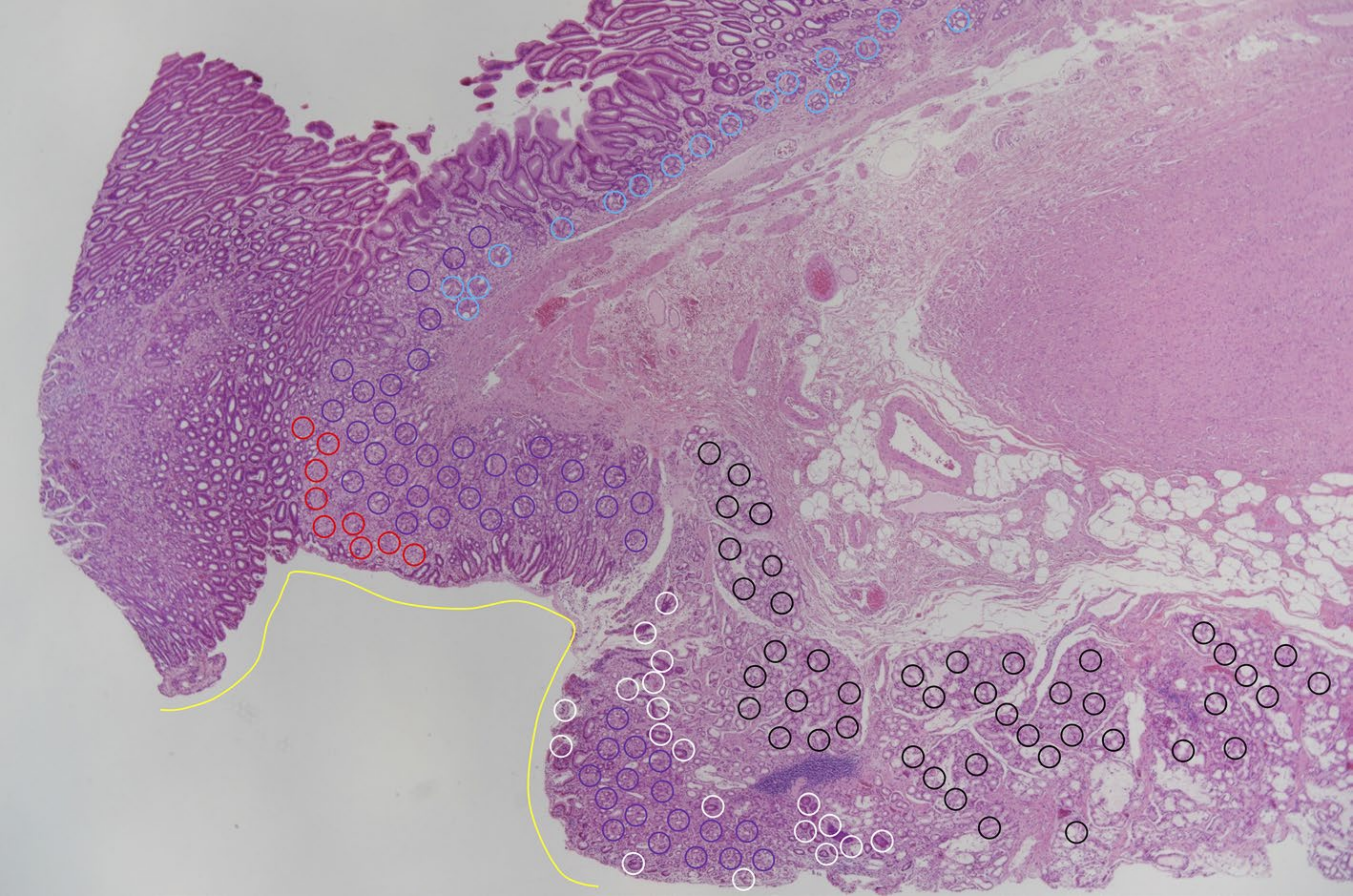
Pathological image of the most antral resection margin. Red circle, signet ring-cell carcinoma; purple circle, fundic glands; light blue circle, pyloric glands; white circle, duodenal epithelium; black circle, Brunner’s gland; yellow line, gastric foveolar epithelium

**Fig. 6:**
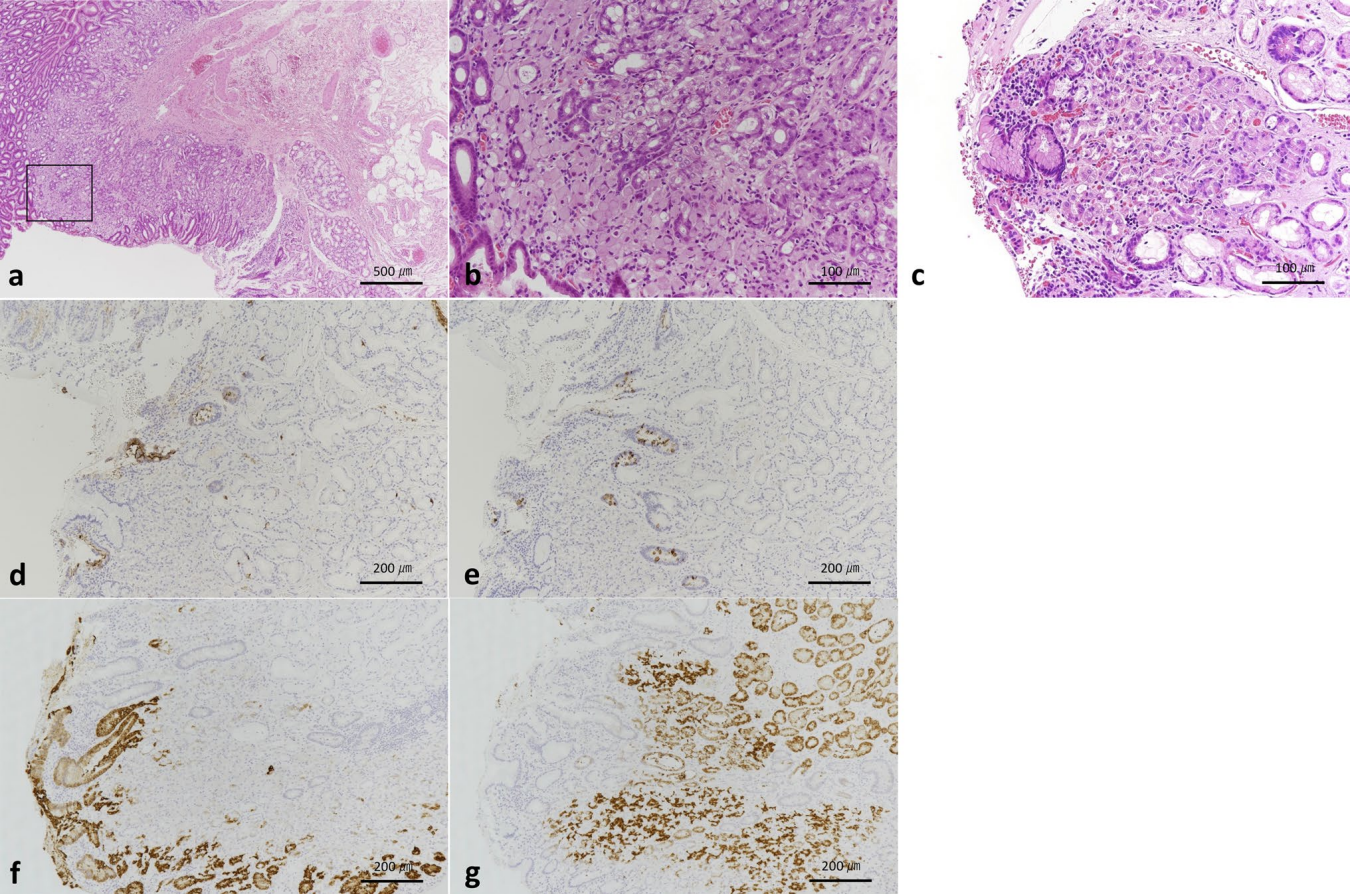
Pathological images of the surgical specimen. **a** Magnified view of the most distal region of the surgical specimen (Fig. 4, red arrowhead). **b** Higher magnification of the boxed area in **a**, showing a signet-ring cell carcinoma (SRCC) surrounded by gastric fundic glands and gastric foveolar epithelium. **c** Duodenal mucosa with ectopic gastric mucosa. **d**–**g** SRCC adjacent to CD10-positive cells **d**, MUC2-positive cells **e**, MUC5AC-positive cells **f** and MUC6-positive cells **g**, suggesting the presence of ectopic gastric mucosa in the duodenum

The anatomical junction between the stomach and the duodenum showed ectopic gastric mucosa extending distally from the gastric pyloric glands (Fig. 5), and the most distal SRCC focus (red arrowhead in Fig. 4) was located within this region. On the anal side of this SRCC, the background consisted of small intestinal mucosa composed of CD10-positive absorptive epithelial cells, MUC2-positive goblet cells, and MUC6-positive Brunner’s glands. Adjacent to this area, ectopic gastric fundic mucosa was identified, containing MUC6-positive fundic glands composed of parietal, chief, and accessory cells, as well as MUC5AC-positive gastric foveolar epithelium (Fig. 6). Although no small intestinal epithelial features were observed within the SRCC lesion itself, the SRCC was clearly located in the duodenal bulb. Based on these findings, it was suggested that this SRCC lesion originated from ectopic gastric mucosa in the duodenum. Retrospective review of preoperative endoscopic images did not reveal detailed visualization of the area in which the SRCC was detected. In addition, no ectopic gastric mucosa was endoscopically identified in other areas of the duodenal bulb (Fig. 1e).

Recognizing the possibility of carcinogenesis from ectopic gastric mucosa in HDGC, we screened the esophagus postoperatively using EGD and evaluated Meckel’s diverticulum with 99mTcO₄⁻ scintigraphy, confirming the absence of ectopic gastric mucosa in both sites. Currently, two years have passed since the surgery, and the patient remains recurrence-free.

**Table 1:** Cases of early hereditary diffuse gastric cancer undergoing total gastrectomy with documented endoscopic and pathological findings.

	Author (year)	Reference no.	Age / sex	Symptoms	H.pylori infection status	Family history of GC	Germline CDH1 mutation	Timing of mutation identification	Site(s) of preoperatively detected lesion(s)	Number of SRCC foci and depth of invation (postoperative pathology)
1	Sugimoto (2014)	6	41/male	No	uninfected	None	Large deletion involving exon 11	postoperative	Antrum, Body	90, M
2	Yamada (2014)	5	55/female	No	uninfected	Father, Grandfather, Son	Large deletion spanning exons 7–16	postoperative	Body	Approximately 200, M
3	Yamada (2014)	5	55/female	No	Not specified	Mother, Grandfather	Large deletion spanning exons 7–16	postoperative	Antrum	32, M
4	Funakoshi (2019)	17	27/female	No	uninfected	Father, Grandmother, Brother	Nonsense mutation in exon 3	postoperative	Body	3, M
5	Hirakawa (2020)	10	25/male	No	Not specified	Father, Brother, Sister	Missense mutation in exon 11	postoperative	Body	36, M
6	Iwaizumi (2020)	18	24/male	Upper abdominal pain	uninfected	Mother	Nonsense mutation in exon 5	postoperative	Body	10, M
7	Namikawa (2021)	16	34/male	Sore throat	Not specified	Not specified	Frameshift mutation in exon 5	preoperative	Antrum	42, M
8	Inaba (2024)	19	41/male	Heartburn	uninfected	Sister, Uncle	Frameshift mutation (exon not specified)	preoperative	Antrum	7, M
9	Present case	–	48/male	No	uninfected	Mother, Grandmother	Missense mutation in exon 14	preoperative	Antrum, Fornix	22, M

*H.pylori*, *Helicobacter pylori*; GC, gastric cancer; SRCC, signet ring-cell carcinoma; M: mucosa.

The patient’s sibling was provided with information regarding genetic counseling but declined it. In addition, the patient’s two minor children are scheduled to undergo genetic counseling after reaching adulthood.

## Discussion

The IGCLC guidelines for HDGC were updated in 2020, with changes to the criteria for recommending genetic testing, primarily by relaxing the age requirement. For example, the threshold for DGC diagnosis was adjusted from under 40 years of age to under 50 years of age. Although this patient had a family history of two cases of gastric cancer, histological confirmation was not obtained. However, the fact that the patient was diagnosed with DGC before the age of 50 met the revised criteria for genetic testing. Ideally, genetic testing should have been proposed when gastric cancer was detected for the second time. However, HDGC was not considered at that point, partly because recurrence of ESD-eligible lesions is not uncommon in Japan.

Table summarizes cases of genetically confirmed early HDGC treated with total gastrectomy, with detailed endoscopic and pathological findings documented. All reported cases originated from Japan, and *CDH1* was the causative gene mutation in all instances. Reports in which genetic mutations were identified preoperatively have increase since 2020, suggesting growing awareness of this disease. Furthermore, the most frequently reported preoperatively identified lesion sites were the antrum and gastric body; notably, the present case was the only one in which a lesion was identified in the fornix.

The R732Q variant identified in this case is a non-conservative amino acid substitution, classified as a missense mutation located in the intracellular domain of E-cadherin [3]. Although missense mutations often include variants of uncertain significance [2], R732Q has been shown to result in loss of E-cadherin function in vitro [3] and is a recurrent mutation identified in multiple families fulfilling the diagnostic criteria for HDGC [4]. In the present case, reduced E-cadherin expression was confirmed by immunohistochemical staining of the carcinoma. Routine immunohistochemical evaluation of E-cadherin expression may be useful for detecting synchronous or metachronous recurrence of SRCC.

The natural history of HDGC remains unclear. However, there is a report of a Japanese woman diagnosed with recurrent gastric cancer 21 years after pyloric gastrectomy [5], suggesting that metachronous recurrence can occur over a long-term course in *CDH1* mutation carriers. In our case, multiple lesions were detected again after the second ESD. Although these lesions likely developed by the time of the second ESD, the fornix lesion was not observed during semiannual EGD follow-up for five years after the first ESD and was therefore considered a metachronous recurrence. Similarly, Sugimoto et al. reported a case in which multiple SRCC lesions were detected two months after ESD [6], However, given the short interval until recurrence, these lesions were likely present at the time of the initial diagnosis. Both cases and our case were negative for *H.pylori*, and while *H. pylori*-negative cases may progress more slowly [7], *H. pylori* infection is known to promote gastric carcinogenesis through methylation of specific gene promoters [8]. It has also been linked to *CDH1* gene methylation [8, 9]. In HDGC patients with *H. pylori* infection, inactivation of the *CDH1* gene may be more pronounced, often leading to advanced-stage cancer. However, Hirakawa et al. reported cases of progressive HDGC in *H. pylori*-negative patients [10], indicating that further studies are needed to determine which genetic mutations contribute to advanced cancer in the absence of *H. pylori* infection. In Japan, as in Europe and the United States, reports of *H. pylori*-negative gastric cancer are increasing [11, 12], raising the possibility that many undiagnosed HDGC cases exist.

The most distal SRCC lesion was considered a carcinoma originating from ectopic gastric mucosa in the duodenum, as it was containing gastric fundic glands, gastric foveolar epithelium, CD10-positive cells, MUC2-positive cells, and Brunner’s glands. Although pyloric glands extend into the oral side of this cancerous lesion, the area immediately surrounding it is lined primarily by cells derived from fundic glands, indicating that the lesion originated not from pyloric glands within the stomach but from ectopic gastric mucosa in the duodenum. To our knowledge, this is the first reported case of carcinogenesis from ectopic gastric mucosa in a *CDH1* mutation carrier. Although carcinogenesis from ectopic gastric mucosa is considered rare [13], COX-2 expression is elevated in ectopic gastric mucosa of the esophagus, independent of *H. pylori* infection or gastric acid exposure [14], suggesting a potential role in tumorigenesis. In experiments using rat intestinal cell lines, COX-2 overexpression led to reduced E-cadherin expression [15], which may synergistically promote tumorigenesis alongside changes caused by *CDH1* mutations. When HDGC is suspected, EGD evaluation of the esophagus and duodenum, along with scintigraphic screening for ectopic gastric mucosa, may be beneficial. Retrospective analysis of the preoperative endoscopic images in this case did not yield detailed visualization of the cancerous area in the duodenum, leaving its characteristic endoscopic features unclear. Given the macroscopic morphology of a 0–IIb lesion covered by foveolar epithelium, which is commonly observed in gastric SRCC, it may be important to initially focus on the presence of ectopic gastric mucosa.

The HDGC guidelines recommend targeted and random biopsies as part of surveillance endoscopy for patients with *CDH1* gene mutations, specifying 28–30 random biopsies. Recently, a case report highlighted the usefulness of magnifying endoscopy in HDGC detection [16]. In our case, SRCC was identified through targeted biopsies from areas with irregular vascular patterns observed using magnifying endoscopy. In early-stage HDGC, multiple lesions are often present. Therefore, if even a single SRCC is found, performing a more detailed examination with magnifying endoscopy may be beneficial.
